# Relationship between *MAN2B1* genotype/subcellular localization subgroups, antidrug antibody detection, and long‐term velmanase alfa treatment outcomes in patients with alpha‐mannosidosis

**DOI:** 10.1002/jmd2.12349

**Published:** 2022-11-25

**Authors:** Line Gutte Borgwardt, Ferdinando Ceravolo, Giulia Zardi, Andrea Ballabeni, Allan Meldgaard Lund

**Affiliations:** ^1^ Department of Paediatrics and Adolescent Medicine Centre for Inherited Metabolic Diseases, Rigshospitalet Copenhagen Denmark; ^2^ Center for Genomic Medicine Copenhagen University Hospital, Rigshospitalet Copenhagen Denmark; ^3^ Chiesi Farmaceutici S.p.A Parma Italy; ^4^ CROS NT S.r.l Verona Italy; ^5^ Department of Clinical Genetics, Centre for Inherited Metabolic Diseases Copenhagen University Hospital, Rigshospitalet Copenhagen Denmark; ^6^ European Reference Network for Hereditary Metabolic Disorders (MetabERN) Udine Italy

**Keywords:** alpha‐mannosidosis, antidrug antibody, infusion‐related reactions, *MAN2B1*, velmanase alfa

## Abstract

Alpha‐mannosidosis (AM), an autosomal recessive disorder caused by pathogenic biallelic variants in the *MAN2B1* gene, leads to lysosomal alpha‐mannosidase deficiency and accumulation of mannose‐rich oligosaccharides. Velmanase alfa (VA), a recombinant human lysosomal alpha‐mannosidase, is the first enzyme replacement therapy for non‐neurological symptoms of AM. Previously, a potential relationship was identified between three *MAN2B1* genotype/subcellular localization subgroups (G1, G2, and G3) and AM disease severity. In VA‐treated patients with AM, it is unknown if a relationship exists between *MAN2B1* genotype/subcellular localization subgroups, antidrug antibodies (ADAs), and infusion‐related reactions (IRRs). This pooled analysis evaluated data from 33 VA‐treated patients with AM to investigate this relationship. Overall, 10 patients were positive for ADAs, 4 of whom had treatment‐emergent ADAs (G1: 3/7 [43%]; G2: 1/17 [6%]; G3: 0/9). Treatment‐emergent ADA‐positive patients with relatively high titers (*n* = 2; G1: 1012 U/ml and G2: 440 U/ml) experienced mild/moderate IRRs that were well‐managed; patients with lower titers (*n* = 2) experienced no IRRs. Overall, changes from baseline in serum oligosaccharides and immunoglobulin G levels did not vary between ADA‐positive and ADA‐negative patients, suggesting a similar effect of VA treatment regardless of ADA status in most patients. Clinical outcomes (3MSCT and 6MWT) were also similar in most patients regardless of ADA status. While further studies are needed, these data suggest a relationship between *MAN2B1* genotype/subcellular localization subgroups and ADA development, with G1 and G2 subgroups more likely to develop ADAs and IRRs. Regardless, this study suggests that ADAs have limited effect on the clinical impact of VA in most patients with AM.

**SYNOPSIS**

Our analysis suggests a relationship between *MAN2B1* genotype/subcellular localization subgroups and the development of antidrug antibodies against velmanase alfa, but it further shows that there appears to be little clinical effect associated with the presence of antidrug antibodies in most velmanase alfa‐treated patients with alpha‐mannosidosis.

## 1 INTRODUCTION

Alpha‐mannosidosis (AM) is a progressive, autosomal recessive disorder caused by pathogenic biallelic variants of the *MAN2B1* gene located on chromosome 19.^1^, ^2^ These variants cause a deficiency in lysosomal alpha‐mannosidase that prevents the degradation of glycoproteins and mannose recycling, causing an accumulation of mannose‐rich oligosaccharides systemically.^1^, ^2^ This accumulation is toxic, impairs cell function, and induces apoptosis.^1^ Clinical presentation varies from mild to severe with a range of symptoms.^3^ The estimated prevalence of AM is 1 in 500 000 people in the general population.^3^ Clinical signs and symptoms include motor function disturbances, intellectual disability, speech and hearing impairments, coarsening of facial features, recurrent infections, skeletal abnormalities, weakness, and ocular changes.^1^, ^2^ While many patients with AM live to be older than 50 years, the long‐term prognosis is poor, with a slow progression of neuromuscular and skeletal deterioration over several decades making most patients wheel‐chair dependent and unable to be socially independent.^2^

Velmanase alfa (VA), a recombinant form of human lysosomal alpha‐mannosidase developed for intravenous (IV) use, is the first approved enzyme replacement therapy (ERT) for the treatment of the non‐neurological symptoms of AM.^4^ ERTs are often associated with immune responses to the exogenous enzyme owing to incomplete immune tolerance and the development of antidrug antibodies (ADAs) that may result in a loss of treatment efficacy and induction of immune complex‐related hypersensitivity reactions.^5^ Cross‐reactive immunological material (CRIM) status has been recognized as a prognostic factor in determining clinical outcome of some ERTs.^6^ No data for CRIM status are available for people with AM, however, preliminary data from people with Pompe disease have shown that CRIM status is related to the nature of the genetic variants.^6^

In a previous investigative study, patients with AM were divided into three genotype/subcellular localization subgroups based on *MAN2B1* variant analyses and subcellular localization of the protein.^1^ A potential correlation between the three *MAN2B1* subgroups and the severity of clinical symptoms was identified.^1^ Subgroup 1 (G1) was characterized as having two null variants (nonsense, frameshift, and large truncations) with mutant protein not localized to lysosomes; subgroup 2 (G2) was characterized as having at least one missense variant or in‐frame deletion/duplication of 1–5 amino acids with mutant protein not localized to lysosomes but instead localized to the endoplasmic reticulum; and subgroup 3 (G3) was characterized as having at least one missense variant or in‐frame deletion/duplication of 1–5 amino acids with mutant protein localized to the lysosome.^1^ Patients in the G1 and G2 subgroups presented with the most severe phenotype, while patients in the G3 subgroup had at least one variant that allowed localization of the mutant MAN2B1 protein to the lysosome and, thus, presented with the mildest phenotype.^1^

In patients with AM treated with VA, it is unknown if there is a relationship between the three *MAN2B1* subgroups and the detection of ADAs and infusion‐related reactions (IRRs). The objective of the present analysis was to evaluate a possible relationship between the three *MAN2B1* subgroups, ADA response dynamics, and IRRs in patients with AM treated with VA.

## 2 METHODS

### 2.1 Pooled analysis design

**FIGURE 1:**
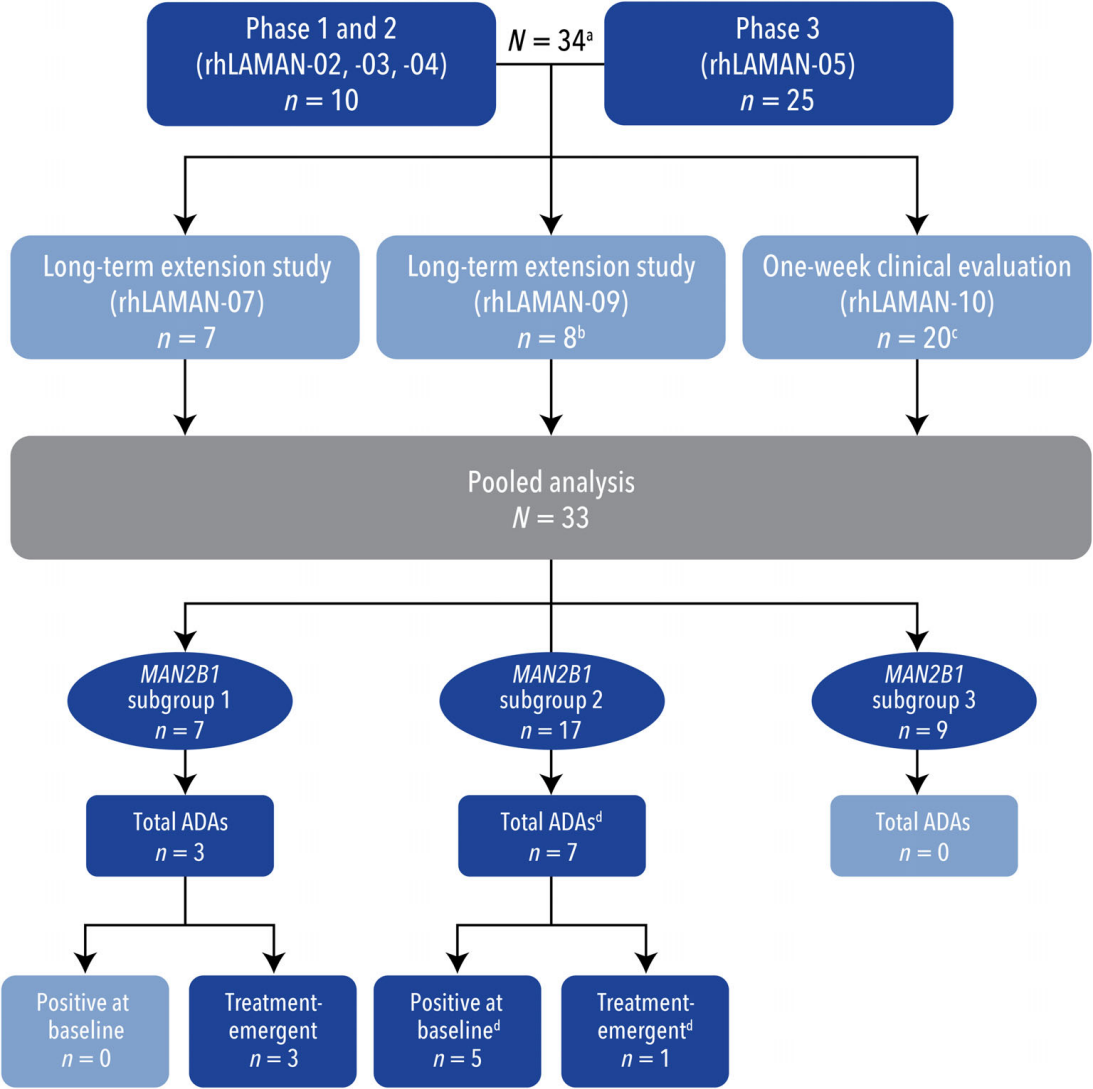
Patient‐flow diagram. (a) One patient participated in rhLAMAN‐02/‐03 and later participated in rhLAMAN‐05. This patient was only counted once. (b) One patient received four doses in rhLAMAN‐09 and then transferred to the compassionate‐use program and participated in the one‐week clinical evaluation. This patient was only counted once. (c) One patient participated in rhLAMAN‐05 in the placebo arm. They then entered the compassionate‐use program and discontinued shortly. As this patient had no data collected during the active treatment period, the patient was excluded from the integrated analysis. (d) One patient had ADA‐positive levels during placebo treatment but not at baseline or during treatment with VA.ADA, antidrug antibody; VA, velmanase alfa

This is a pooled analysis of seven clinical trials that included evaluation of clinical immunogenicity in pediatric and adult VA‐treated patients with AM (Figure 1). Patient data were obtained and collated from phase 1/2 clinical trials (rhLAMAN‐02^7^ [NCT01268358^8^], rhLAMAN‐03 [NCT01285700^9^], rhLAMAN‐04 [NCT01681940^10^]), a phase 3 clinical trial (rhLAMAN‐05^11^ [NCT01681953^12^]), the subsequent extension trials (rhLAMAN‐07 [NCT01908712^13^] and rhLAMAN‐09 [NCT01908725^14^]), and a one‐week clinical evaluation on patients from the compassionate‐use program (rhLAMAN‐10 [NCT02478840^15^]). Patients were eligible for this pooled analysis if they had a confirmed diagnosis of AM, had been or were actively participating in one of the aforementioned trials, and were receiving weekly IV infusions of VA or placebo at the time of data collection. All patients underwent biochemical and clinical assessments at baseline, at prespecified time points, and at last observation based on the trial in which they were enrolled. Patients enrolled in rhLAMAN‐10 underwent a one‐week clinical evaluation and were assessed in a single, centralized, and comprehensive evaluation visit. Patients from rhLAMAN‐10 attended a screening visit on day 1 where, after an initial evaluation, they received their scheduled weekly dose of VA 1 mg/kg body weight by IV infusion.

ADA formation was monitored at regular intervals during treatment using a validated ADA enzyme‐linked immunosorbent screening assay that co‐incubated patient serum samples and adsorbed Protein G to measure the binding of sample immunoglobulin G (IgG) to Protein G. The bound sample IgG was then detected by sequential addition of biotinylated VA and streptavidin‐HRP. The optical density of the well positively correlated to the ADA level of the sample. ADA levels were expressed in terms of U/ml relative to a calibration curve established using a rabbit anti‐VA IgG‐positive control. Assay sensitivity was approximately 1.0 U/ml serum, corresponding to 100 ng/ml of the positive control IgG. Samples were tested in a single stage where all samples underwent an ADA screening assay and were classified as negative (<1.4 U/ml) or positive (≥1.4 U/ml). Samples from patients who experienced IRRs were further tested in a nonvalidated assay to detect antidrug immunoglobulin E (IgE) antibodies.

Patients were classified into one of three *MAN2B1* subgroups based on variant analyses and subcellular MAN2B1 protein localization as described in Riise Stensland et al.^1^, ^16^ Briefly, variant analyses were conducted through sequencing of the 24 *MAN2B1* exons, corresponding exon‐intron boundaries, and parts of the 5′ and 3′ untranslated regions using the Sanger method.^1^ When possible, parents were also analyzed for variants found in their children to confirm carrier status and allelic phase of the variants.^1^ To determine subcellular MAN2B1 protein localization of each patient genotype, each genotype was reproduced in vitro via site‐directed mutagenesis, carried out on wild‐type *MAN2B1* cDNA on a pcDNA 3.1 vector. Protein localization was determined in transfected HeLa cells via immunocytochemistry and confocal microscopy.^1^ CRIM status at baseline was not measured because of the unavailability of a suitable assay.

The source clinical trials were all conducted in accordance with the Declaration of Helsinki (1964 and amendments) and current ICH good clinical practice guidelines. All patients or parents/legal guardians provided written informed consent. All participating sites obtained independent ethics committee/institutional review board approval.

### 2.2 Pooled analysis outcomes

The main objective of this pooled analysis was to evaluate the relationship between the *MAN2B1* subgroups, pretreatment and treatment‐emergent ADAs, serum oligosaccharide levels, total serum IgG levels, and IRRs in patients with AM treated with VA for up to 48 months. Relevant descriptive outcomes included ADA‐positive/negative status, ADA levels at different times prior to and during treatment, impact of ADA formation on drug exposure, pharmacodynamic response versus ADA level versus time, relationship of ADAs to clinical outcomes, and incidence of treatment‐emergent ADAs versus *MAN2B1* subgroup. Additional safety outcomes included frequency of IRRs, frequency of IRRs by *MAN2B1* subgroup, and IRR severity, and patient outcomes.

## 3 RESULTS

### 3.1 Pooled analysis population

Data from 33 patients (14 adults [≥18 years] and 19 pediatric patients [<18 years]) with up to 48 months of VA exposure were included in this pooled analysis (Table S1). All 33 patients (100%) were exposed to VA treatment for a minimum of 6–12 months with 27.3% of patients having 36–48 months of VA exposure. Baseline demographics and characteristics of study participants are detailed in Table 1. The mean (standard deviation [SD]; range) age of patients was 17.1 (7.8; 6–35) years, and 60.6% of patients were male. The mean (SD) residual alpha‐mannosidase activity of patients at baseline was 4.5% (1.6). Residual activity may be due to other non‐lysosomal alpha‐mannosidases expressed in cells such as Golgi alpha‐mannosidase and cytosolic alpha‐mannosidase.

**TABLE 1:** Baseline demographics and characteristics

Parameter		Pooled analysis group (*N* = 33)
Age (years)	Mean (SD)	17.1 (7.8)
	Median (minimum; maximum)	15.0 (6.0; 35.0)
Race (*n*, %)	White	33 (100.0)
Sex (*n*, %)	Male	20 (60.6)
	Female	13 (39.4)
Weight (kg)	Mean (SD)	58.8 (18.6)
	Median (minimum; maximum)	65.0 (18.7; 95.2)
Height (m)	Mean (SD)	1.53 (0.18)
	Median (minimum; maximum)	1.57 (1.12; 1.81)

*Note*: Unless otherwise specified, percentages were based on the number of patients with available data and were not calculated for missing categories.

Abbreviation: SD, standard deviation.

### 3.2 Pooled analysis outcomes

#### 3.2.1 Detection of ADAs and ADA levels

**FIGURE 2:**
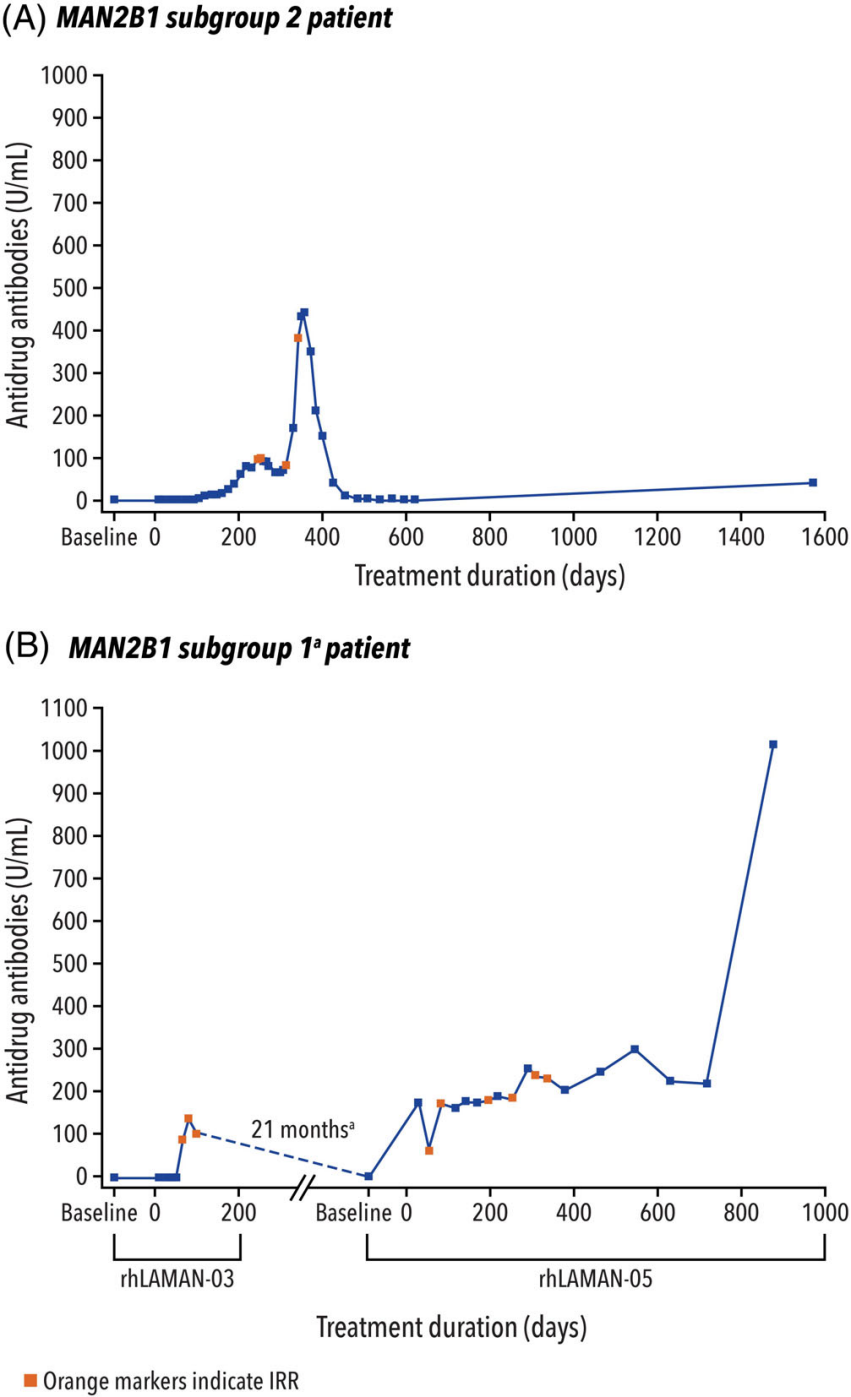
ADA levels over time for the two patients with high‐ADA levels (and by *MAN2B1* subgroup). (a) The patient who experienced high‐ADA levels in *MAN2B1* subgroup 1 was initially enrolled in rhLAMAN‐03 but was discontinued due to adverse events (anaphylactoid reaction). After 21 months of time off‐treatment, the patient subsequently enrolled in rhLAMAN‐05 study and was randomized to the active‐treatment arm during which time the high‐ADA levels were recorded.ADA, antidrug antibody; AE, adverse event; IRR, infusion‐related reaction

Overall, 10/33 (30.3%) patients were ADA‐positive at ≥1 timepoint during the analysis (Tables S2 and S3). Five of the 10 (50.0%) patients were confirmed as ADA‐positive at baseline/pretreatment (referring to drug‐reactive antibodies present in treatment‐naïve patients, which may be due to different etiologies, including prior exposure to structurally similar lysosomal proteins and/or endogenous antibody cross‐reactivity), with ADA levels ranging from 1.4 to 3.1 U/ml. These five patients had relatively low‐ADA levels throughout the trials, with maximum levels ranging from 2.0 to 4.9 U/ml. Most of these patients experienced a decrease in ADA level after the maximal value was reached. One of the 10 (10%) patients had ADA‐positive results during placebo treatment but not at baseline or during treatment with VA. Four of the 10 (40.0%) patients had treatment‐emergent ADAs, defined as ADA‐negative at baseline but ADA‐positive at ≥1 on‐treatment sample timepoint. These four patients expressed a mean residual enzyme activity of 3.20% compared with 4.66% for the remaining 29 patients (4.49% for the 5 patients that were ADA‐positive at baseline), potentially demonstrating a relationship between lower residual enzyme activity and the development of ADAs with VA treatment. Of these four patients, two had high‐ADA levels of >80 U/ml. These two patients experienced maximal ADA levels of 440 U/ml and 1012 U/ml, respectively, and both patients experienced IRRs (Figure 2).

### 3.3 ADAs by MAN2B1 subgroups

All 33 patients were sorted into three *MAN2B1* subgroups based on *MAN2B1* variant analysis and subcellular localization of the protein (Figure 1). The G1 subgroup consisted of seven patients (mean [SD] residual enzyme activity: 3.97% [0.86]). The G2 subgroup consisted of 17 patients (mean [SD] residual enzyme activity: 4.94% [2.05]). The G3 subgroup consisted of nine patients (mean [SD] residual enzyme activity of 4.21% [0.95]).

Of the 10 patients with ADAs, 3/7 (43%) patients were in the G1 subgroup, 7/17 (41%) patients in the G2 subgroup, and 0/9 patients in the G3 subgroup. Treatment‐emergent ADAs were detected in four patients: 3/7 in the G1 subgroup and 1/17 in the G2 subgroup. Of these four patients, one patient in the G1 subgroup experienced a high‐ADA level of 1012 U/ml and one patient in the G2 subgroup experienced a high‐ADA level of 440 U/ml (Figure 2). All patients that were ADA‐positive at baseline were in the G2 subgroup (6/17).

### 3.4 Serum oligosaccharide and IgG levels

**FIGURE 3:**
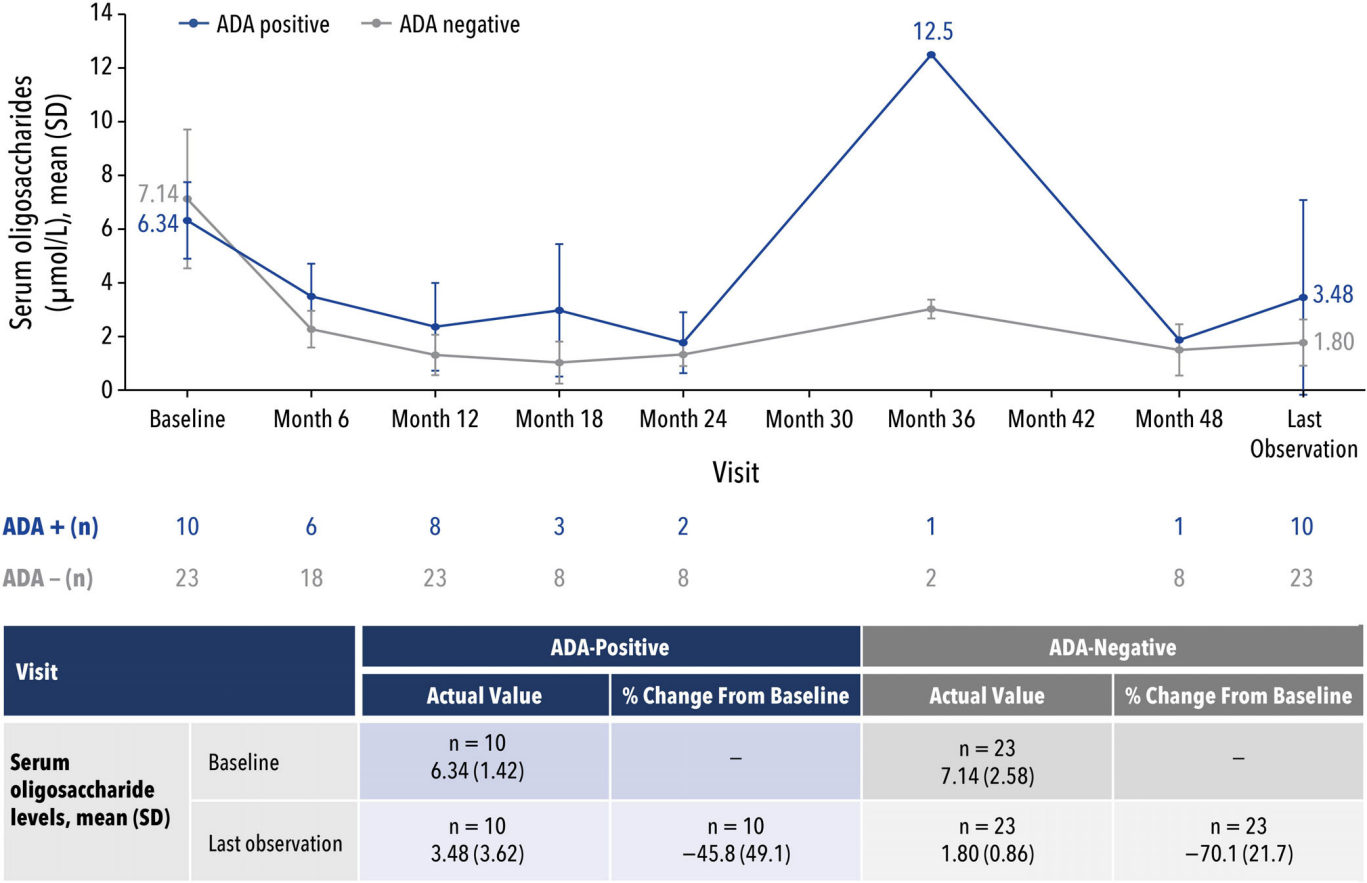
Serum oligosaccharide levels by ADA status.ADA, antidrug antibody; SD, standard deviation

**FIGURE 4:**
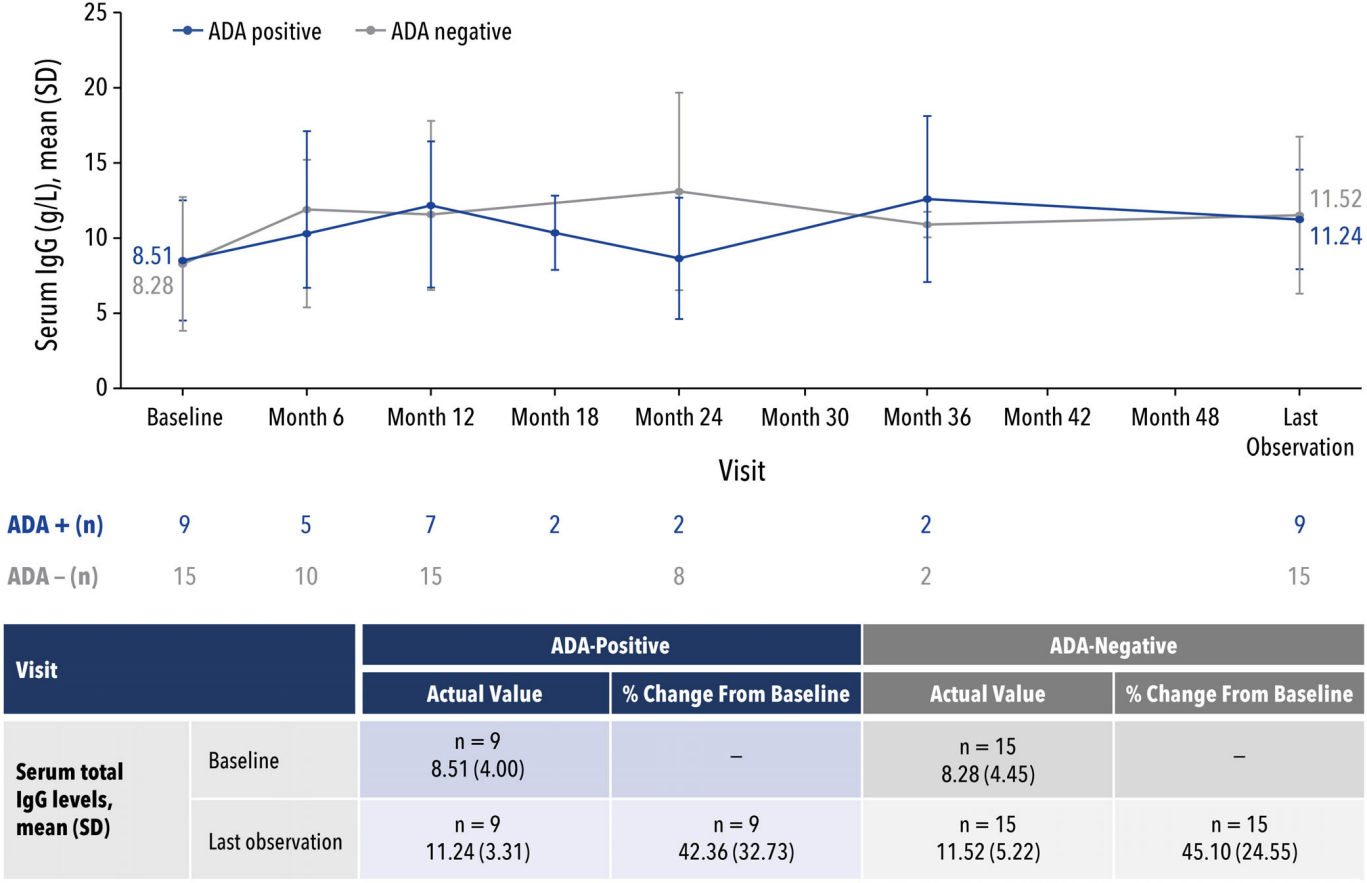
Serum IgG levels by ADA status.ADA, antidrug antibody; IgG, immunoglobulin G; SD, standard deviation

In patients with AM, decreased alpha‐mannosidase activity results in increased serum oligosaccharide levels, and an impaired immune response with decreased serum IgG levels. Thus, a decrease in serum oligosaccharides and an increase in IgG levels can be indicative of successful treatment in patients with AM and were evaluated in this pooled analysis. The relationships between ADA status and change from baseline in serum oligosaccharide and IgG levels are presented in Figures 3 and 4, respectively.

For serum oligosaccharide levels, the mean percent change (SD) from baseline to last observation was −45.8% (49.1) for ADA‐positive patients (*n* = 10) and 70.1% (21.7) for ADA‐negative patients (*n* = 23) (Figure 3). A sensitivity analysis (Figure S1) using a simple linear regression model was performed to understand if any specific patients influenced the difference in mean percent change between ADA‐positive and ADA‐negative results. Through an iterative process, a total of three ADA‐positive patients, including one with an ADA‐high level of 1012 U/ml, potentially influenced the data. Before eliminating any of these three patients, there was a marginal statistically significant difference in percent change from baseline in serum oligosaccharides in the ADA‐positive and ADA‐negative groups (*p* = 0.05). When any single patient was removed, there was no statistically significant difference between the ADA‐positive and ADA‐negative groups (*p* = 0.21, 0.64, and 0.60 for the removal of each subsequent patient).

For serum IgG levels, the increase in mean percent change (SD) from baseline to the last observation was similar for 9 ADA‐positive patients compared with 15 ADA‐negative patients (42.4% [32.73] vs. 45.1% [24.55]; *p* = 0.82) (Figure 4). Regardless, the same approach was used to identify potential outliers in the IgG analysis. This analysis revealed a single ADA‐positive patient to have potentially influenced the data. Note that this patient was not one of the three influential patients in the serum oligosaccharide analysis. Removing this patient did not change whether ADA status significantly affected the change in serum IgG levels; there was no significant difference between the ADA‐positive and ADA‐negative groups (*p* = 0.27).

### 3.5 Relationship of ADA to drug levels and serum oligosaccharides

**FIGURE 5:**
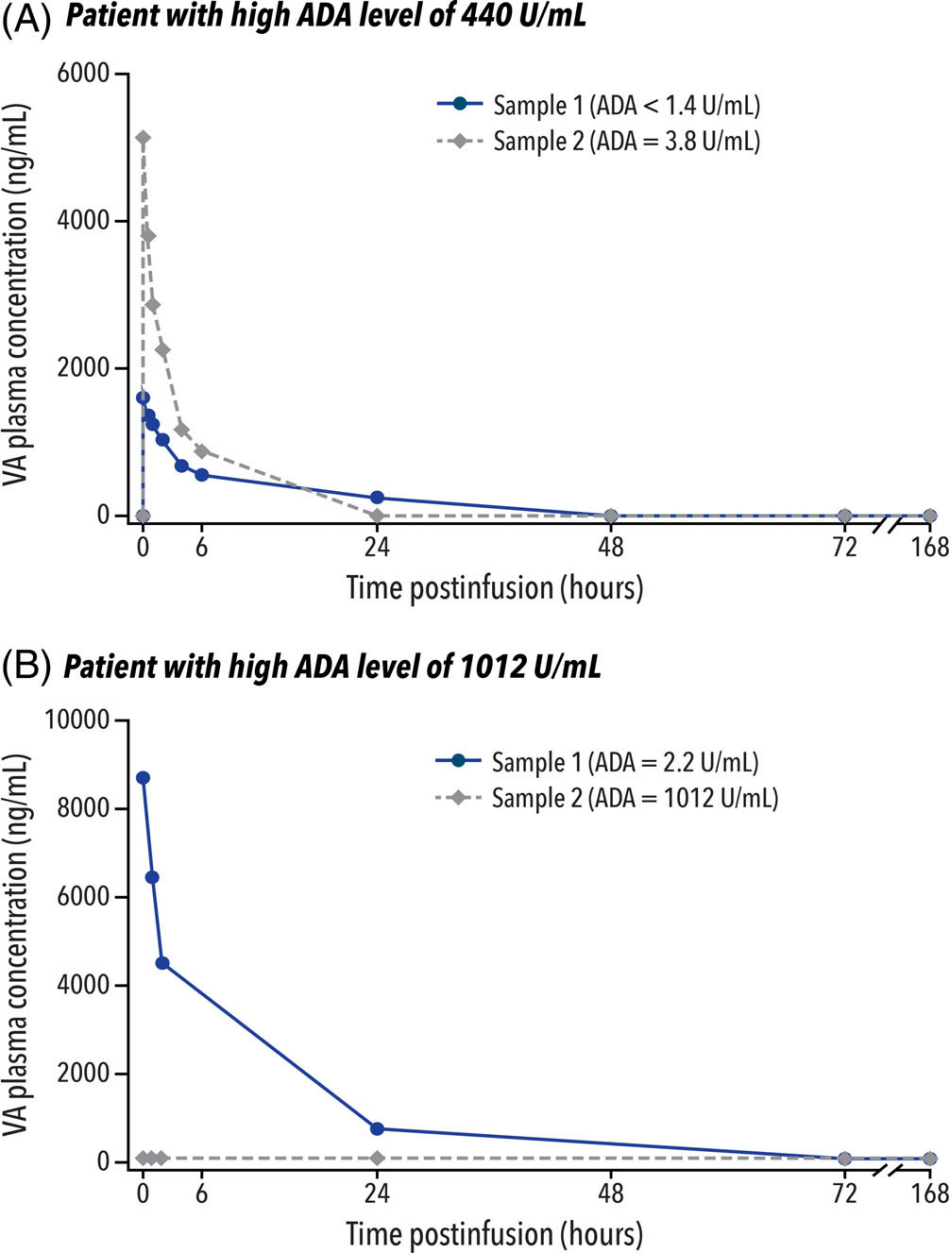
Relationship of ADA to drug levels for the two patients with high‐ADA levels. (a) Sample 1 for both patients was taken immediately after administration of the first dose of VA. ADA, antidrug antibody; VA, velmanase alfa

Pharmacokinetic parameters were evaluated for two patients who developed treatment‐emergent ADAs; these two patients developed high‐ADA levels of 440 and 1012 U/ml. No other ADA‐positive patients had pharmacokinetic data available for both before and after seroconversion. For the patient with a high‐ADA level of 440 U/ml, VA plasma concentration samples were collected at two timepoints, with an ADA level of <1.4 U/ml (ADA‐negative) for the first sample and an ADA level of 3.8 U/ml (ADA‐positive) for the second sample. For this patient, the detection of ADAs did not affect VA plasma concentrations, which were higher following the second sample compared with those measured following the first sample (Figure 5A). For the patient with a high‐ADA level of 1012 U/ml, VA plasma concentration samples were also collected at two timepoints, with ADA levels of 2.2 U/ml (ADA‐positive) for the first sample and 1012 U/ml (ADA‐high) for the second sample. This patient showed no quantifiable VA plasma concentration at any timepoint during the ADA‐high sample evaluation (Figure 5B). It can most plausibly be explained by the exceptionally high‐ADA level potentially causing the development of antibodies that fully bound VA in the plasma, thereby preventing detection in the assay used to measure VA.

The relationship between the development of ADAs and serum oligosaccharide levels was also evaluated for these two patients with high‐ADA levels (Table 2). For the patient with an ADA‐high level of 440 U/ml, serum oligosaccharides were normalized under treatment, regardless of the development of ADAs. For the patient with an ADA‐high level of 1012 U/ml, however, an initial decrease in serum oligosaccharides was observed up to 12 months, followed by an increase to higher than baseline. In this patient, the increase in serum oligosaccharides corresponded to the increase in ADA level to 1012 U/ml, showing a potential relationship between exceptionally high‐ADA levels and lack of effectiveness of VA during that period.

**TABLE 2:** Relationship of ADA to serum oligosaccharides for patients with ADA‐high levels

Parameter	Time (months)							
	Baseline	0–6	6–12	12–18	18–24	24–30	30–36	36–48
Patient with ADA‐high level of 440 U/ml								
Anti‐VA antibodies (U/ml)	<1.4	25.0	440	<1.4	‐	‐	‐	<1.4
Serum oligosaccharides (μmol/L)								
Absolute value	7.0	5.0	1.0	2.0	‐	‐	‐	1.9
Change from baseline		−2.0	−6.0	−5.0	‐	‐	‐	−5.1
% Change from baseline		−28.6	−85.7	−71.4	‐	‐	‐	−72.9
Patient with ADA‐high level of 1012 U/ml^a^								
Anti‐VA antibodies (U/ml)	2.2	176.4	250.9	299.0	224.0	1012.0	‐	‐
Serum oligosaccharides (μmol/L)								
Absolute value	8.1^a^	4.6	4.0	12.5^b^				‐
Change from baseline		−3.5	−4.1	4.4^b^				‐
% Change from baseline		−43.2	−50.6	54.3^b^				‐

Abbreviations: ADA, antidrug antibody; VA, velmanase alfa.

^a^ This patient participated in the rhLAMAN‐02 and rhLAMAN‐05 studies. Data from rhLAMAN‐02 are not included, and baseline for these data are the start of the rhLAMAN‐05 study.

^b^ Values for 12–36 months.

### 3.6 Relationship of ADA to clinical outcomes

**FIGURE 6:**
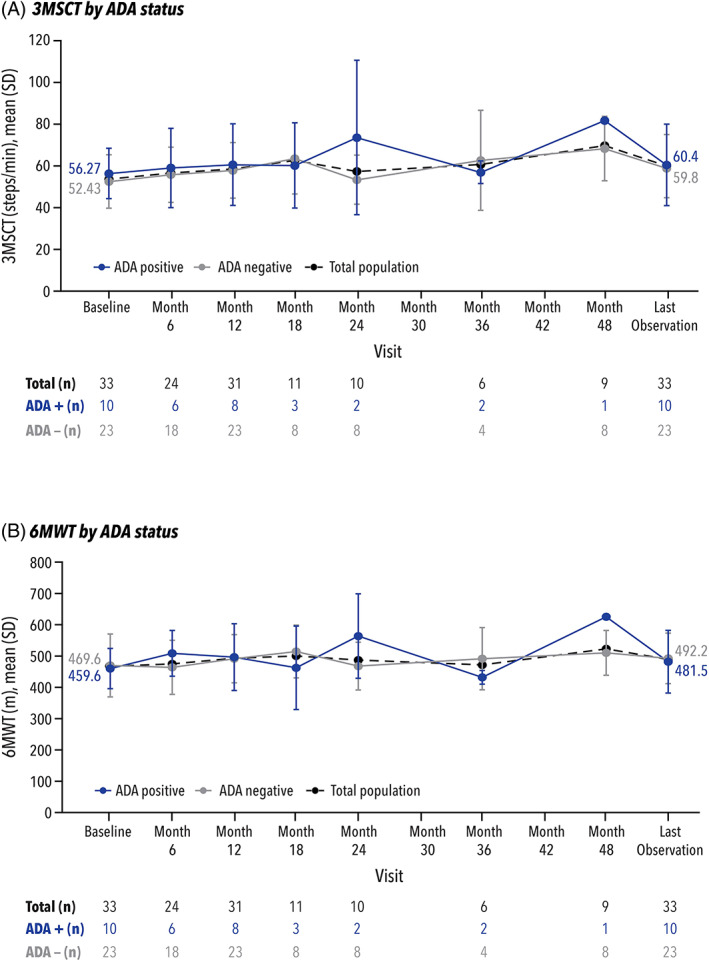
Clinical outcomes (3MSCT and 6MWT) by ADA status. 3MSCT, 3‐minute stair climb test; 6MWT, 6‐minute walk test;ADA, antidrug antibody; SD, standard deviation

All patients underwent clinical assessments, including the 3‐minute stair climb test (3MSCT) and the 6‐minute walk test (6MWT), at prespecified timepoints. For the total population, there was a significant improvement in 3MSCT at 12 months (*n* = 31; mean change: +4.25 steps/min [*p* = 0.01]; mean percent change: +9.3% [*p* = 0.01]), and these findings remained statistically significant at last observation (*n* = 33).^17^ 3MSCT was further evaluated by ADA status (Figure 6A). At baseline, the mean value was 53.60 steps/min for the total population, 52.43 steps/min for the ADA‐negative group (*n* = 23), and 56.27 steps/min for the ADA‐positive group (*n* = 10). At each specified timepoint, the mean steps per minute were similar between the three groups, with differences seen when fewer patients were evaluated. By last observation, 3MSCT improved to 59.98 steps/min in the total population (*n* = 33; mean change: +6.38 steps/min [*p* < 0.01]; mean percent change: +13.8% [p < 0.01]),^17^ 59.80 steps/min in the ADA‐negative group (*n* = 23; mean change: +7.36 steps/min; mean percent change: +17.0%), and 60.40 steps/min in the ADA‐positive group (*n* = 10; mean change: +4.13 steps/min; mean percent change: +6.3%).

For the total population, there was a positive trend towards improvement in 6MWT at 12 months (*n* = 31; mean change: 21.9 meters [*p* = 0.07]; mean percent change: 7.3% [*p* = 0.09]), and the improvement was statistically significant at last observation (*n* = 33).^17^ 6MWT was further evaluated by ADA status (Figure 6B). At baseline, the mean value was 466.6 m for the total population,^17^ 469.6 m for the ADA‐negative group (*n* = 23), and 459.6 m for the ADA‐positive group (*n* = 10). Differences between the two groups were primarily seen at timepoints when fewer patients were evaluated. By last observation, 6MWT improved to 489.0 m in the total population (*n* = 33; mean change: 22.4 m [*p* = 0.05]; mean percent change: +7.1% [*p* = 0.07]),^17^ 492.2 m in the ADA‐negative group (*n* = 23; mean change: +22.6 m; mean percent change: +8.3%), and 481.5 m in the ADA‐positive group (*n* = 10; mean change: +21.9 m; mean percent change: +4.5%).

### 3.7 Safety outcomes

Approximately 2800 VA infusions were performed across all controlled clinical trials included in this pooled analysis, with approximately 2000 VA infusions performed in pediatric patients. In the combined adult and pediatric populations, 1‐in‐147 VA infusions led to an IRR, while in the pediatric population, 1‐in‐105 VA infusions led to an IRR. All IRRs were characterized by a rapid onset of symptoms occurring within 2 h of treatment with VA. All IRRs were mild to moderate in severity, and outcomes were reported as recovered or resolved following standard risk‐management measures including reduction of the infusion rate of VA, and/or premedication with corticosteroids and antihistamines.

Three of 33 (9.1%) patients experienced a total of 19 IRRs, and it was concluded that these events were due to VA administration. Of those three patients, two ADA‐positive patients with high‐ADA levels experienced a total of 18 IRRs (one patient in the G1 subgroup: 14 IRRs; one patient in the G2 subgroup: 4 IRRs) and no VA‐specific IgE was detected in either of the two patients. One ADA‐negative patient (G2 subgroup) experienced only one IRR of mild intensity, which resolved rapidly; this occurred at the first infusion point and was not considered consistent with the profile of a typical IRR likely to occur with VA treatment. Those IRRs that coincided with high levels of serum ADA were most plausibly explained as immune‐complex‐related hypersensitivity reactions.

A total of 546 treatment‐emergent adverse events (TEAEs) were reported by 29 (87.9%) patients. The most frequently reported TEAEs included nasopharyngitis (*n* = 23), headache (*n* = 13), pyrexia (*n* = 11), vomiting (*n* = 10), cough (*n* = 9), and diarrhea (*n* = 9). The majority of TEAEs (99.3%) were of mild to moderate severity and had resolved by the end of the trials.

## 4 DISCUSSION

ERT can often result in the development of ADAs, which may lead to a loss of treatment efficacy and the development of serious adverse events.^5^ An example of this is the lysosomal‐storage disorder Pompe disease, in which ADA‐generation has been shown to interfere with enzyme uptake, inhibit enzyme activity, and correlate with poor outcomes.^18^ In Pompe disease, genetic‐variant data have been shown to aid in predicting the effectiveness of ERT.^6^ Similar data are not available for patients with AM to date; however, a previous study identified three AM subgroups based on a *MAN2B1* variant analysis and the subcellular localization of the protein.^1^

The present pooled analysis evaluated the potential immune‐complex‐related hypersensitivity that can occur in patients with AM who develop ADAs during long‐term administration of VA and assessed its relationship to the *MAN2B1* subgroups through the monitoring of levels of ADAs, serum oligosaccharide, serum IgG, and any IRRs. These parameters were further analyzed by *MAN2B1* subgroups to determine the relationship between the subgroups and the long‐term outcomes of VA treatment in patients with AM.

With up to 48 months of VA exposure, only a small proportion (4 of 33 [12%]) of patients with AM developed treatment‐emergent ADAs. Of these four patients, only two developed high‐ADA levels that were associated with IRRs. No VA‐specific IgE was detected in either of the two patients. Most patients who developed ADAs had very low‐ADA levels and experienced a drop in their ADA levels after a maximal value was reached. Regardless of the development of ADAs or IRRs, the impact of VA—as measured by serum oligosaccharides and serum IgG levels—was maintained in most patients.

In patients with AM, the reduction of serum oligosaccharides and the elevation of serum IgG levels may represent potential beneficial effects of VA and could lead to stabilization of non‐neurological disease progression in this patient population. In this analysis, reduced serum oligosaccharide levels and elevation of serum IgG levels indicate that long‐term VA treatment achieves sustained clinical usefulness.

Further sub‐analysis by *MAN2B1* subgroups revealed a correlation between the development of ADAs and the subgroups. Patients in the G1 and G2 subgroups who were expected to have the most severe phenotypes were also more prone to developing ADAs, with >40% of the patients in each of those subgroups developing ADAs. Furthermore, all treatment‐emergent ADAs were limited to the G1 and G2 subgroups in this analysis. In the G1 subgroup (two null variants; mean residual enzyme activity of 3.97%), 3/7 patients developed treatment‐emergent ADAs; this could potentially be due to the lack of residual expression of the enzyme, causing enzyme replacement to elicit a stronger immune response to exogenous enzyme.^1^ In the G2 subgroup (mean residual enzyme activity of 4.94%), only 1/17 patients developed treatment‐emergent ADAs. In the G3 subgroup (mean residual enzyme activity of 4.21%), no patients developed ADAs; this subgroup was expected to have the mildest clinical presentation since patients had at least one genetic variant that allowed for localization of mutant MAN2B1 protein to lysosomes and, thus, could potentially have some residual activity of lysosomal alpha‐mannosidase, allowing for a milder immune response to exogenous enzyme replacement.^1^

The outcomes assessed were similar between the three *MAN2B1* subgroups, and efficacy of treatment was maintained for patients in each subgroup regardless of ADA detection. For ADA evaluation by *MAN2B1* subgroups, it is worthwhile to note that no patients in the G3 subgroup developed detectable ADAs. Thus, all patients from the G3 subgroup were in the ADA‐negative group for serum oligosaccharide evaluations, causing an unbalanced match in disease phenotype between the ADA‐positive and ADA‐negative groups. Even though the severity of clinical symptoms may have differed between the ADA‐positive and ADA‐negative groups, baseline serum oligosaccharide and serum IgG levels were similar in both groups, and the effect of VA was maintained throughout treatment, as seen with the 3MSCT and 6MWT by ADA status.

Regardless of *MAN2B1* subgroups or development of ADAs, all incidences of IRRs were mild to moderate in severity and were reported as recovered/resolved. High levels of ADAs were only seen in 2 of 33 patients and were associated with an elevated incidence of IRRs. However, in our experience, even in the presence of high‐ADA levels, further IRRs could be prevented or mitigated by premedication with corticosteroids or antihistamines and reduced infusion rates.

### 4.1 Limitations of this analysis

As this study is not a prospective analysis, the conclusions that can be drawn from this pooled analysis lack prospective validation. Further limitations include small sample size; absence of CRIM measurement; and reliance on pharmacodynamic markers, serum oligosaccharide levels, and total serum IgG level as surrogate indices of treatment benefit.

## 5 CONCLUSION

These findings suggest there may be a correlation between genetic variants in *MAN2B1* and ADA development; in this analysis, patients in the G1 and G2 *MAN2B1* subgroups were more likely to develop ADAs and subsequent IRRs. Despite the development of ADAs, IRRs were limited in occurrence, and there did not appear to be large differences in efficacy responses between the *MAN2B1* subgroups. Adverse events such as IRRs were managed through reduction of infusion rates and premedication as necessary. While future studies are required to assess the relationship between *MAN2B1* subgroups and ADA development, this analysis demonstrates that ADAs have a limited effect on the clinical benefit of VA in patients with AM regardless of the *MAN2B1* subgroup and, as such, provides meaningful insight into the disease management of AM in both pediatric and adult patients.

## AUTHOR CONTRIBUTIONS

Line Gutte Borgwardt, Ferdinando Ceravolo, and Allan Meldgaard Lund provided substantial contributions to the conception and design of this analysis. All authors (Line Gutte Borgwardt, Ferdinando Ceravolo, Giulia Zardi, Andrea Ballabeni, Allan Meldgaard Lund) provided substantial contributions to the acquisition, analysis, and interpretation of data for this analysis. All authors contributed to drafting/revising the work and provided final approval of the version to be published. All authors agree to be accountable for all aspects of the work in ensuring that questions related to the accuracy or integrity of any part of the work are appropriately investigated and resolved.

## CONFLICTS OF INTEREST

Line Gutte Borgwardt has received consulting fees from Chiesi, the sponsor of the study; Ferdinando Ceravolo was an employee of Chiesi Farmaceutici S.p.A., the sponsor of the study; Giulia Zardi has received consulting fees from Chiesi Farmaceutici S.p.A., the sponsor of the study; Andrea Ballabeni is an employee of Chiesi Farmaceutici S.p.A., the sponsor of the study; Allan Meldgaard Lund has received consulting fees and/or honoraria/travel support from Amicus, BioMarin, Chiesi, Sanofi Genzyme, Shire/Takeda, Recordati, and Sobi as well as grant/research support from Sanofi Genzyme and Shire/Takeda.

## ETHICS APPROVAL AND PATIENT CONSENT STATEMENT

The study protocol, patient information, and patient informed consent forms were reviewed and approved by an Independent Ethics Committee (De Videnskabsetiske Komiteer Region Hovedstaden; Hillerød, Denmark) and a Regulatory Agency (Danish Medicin Agency; København S, Denmark) complying with the requirements of European Federal regulations and the International Conference on Harmonisation (ICH) before enrollment of patients. This study was conducted in accordance with *The Code of Ethics of the World Medical Association (Declaration of Helsinki 1964, as revised in 2013)*, ICH good clinical practice guidelines, local guidelines, and applicable regulations when developing, obtaining, and documenting the patient informed consent. Informed consent was obtained from all individual participants included in the study.

## ANIMAL RIGHTS

This study does not contain any studies with animal subjects performed by any of the authors.

## Supporting information


**Figure S1** Serum oligosaccharides and serum IgG sensitivity analysis by ADA status
**Table S1** Summary of studies contributing to the pooled analysis
**Table S2** Summary of ADA levels for ADA‐positive samples
**Table S3** ADA‐positive patients by category

## Data Availability

At this time, we will approve or deny data requests from external parties on a case‐by‐case basis. Chiesi reserves the right to deny requests for any and all legally appropriate reasons. Data requests that risk sharing participant‐level data or proprietary information will not be approved.
