# Supplementary material for: Relationship between MAN2B1 genotype/subcellular localization subgroups, antidrug antibody detection, and long‐term velmanase alfa treatment outcomes in patients with alpha‐mannosidosis
Source: JIMD Rep. 2022 Nov 25;64(2):187–98. doi: 10.1002/jmd2.12349 (PMC9981422; doi:10.1002/jmd2.12349)
Supplement: Supplementary file 1 — Figure S1 Serum oligosaccharides and serum IgG sensitivity analysis by ADA status Table S1 Summary of studies contributing to the pooled analysis Table S2 Summary of ADA levels for ADA‐positive samples Table S3 ADA‐positive patients by category [file JMD2-64-187-s001.docx]

**Supplementary Tables/Figures:**

**Supplemental Table 1:** Summary of studies contributing to the pooled analysis.

| **Clinical Trial ID** | **Number of Patients** | **Dose Level of VA** | **Study Duration** |
| --- | --- | --- | --- |
| **rhLAMAN-02** | 2  2  2  2  2 | 6.25 U/kg  12.5 U/kg  25 U/kg  50 U/kg  100 U/kg | 5 weeks  4 weeks  3 weeks  2 weeks  1 week |
| **rhLAMAN-03** | 5  5^a^ | 25 U/kg  50 U/kg | 52 weeks  52 weeks |
| **rhLAMAN-04** | 9 | 1 mg/kg | 32–87 weeks |
| **rhLAMAN-05** | 15  10 | 1 mg/kg  Placebo | 52 weeks  52 weeks |
| **rhLAMAN-07^b^** | 7 | 1 mg/kg | ongoing |
| **rhLAMAN-09^b^** | 8^c^ | 1 mg/kg | ongoing |
| **rhLAMAN-10** | 20^c,d^ | 1 mg/kg | 1 week |

ADA, antidrug antibody; VA, velmanase alfa.

^a^One patient withdrew after 9 infusions.

^b^rhLAMAN-07 and rhLAMAN-09 study results were included in the integrated analysis with the cutoff date of 11 April 2016; these studies are still ongoing.

^c^One patient received 4 doses of VA in rhLAMAN-09 before transferring to the compassionate-use program and being included in rhLAMAN-10.

^d^Twenty patients participated in the compassionate-use program but only 18 patients were involved in the rhLAMAN-10 data collection. Regardless, 19 of the 20 patients were included in the integrated analysis. One patient was excluded because no data were collected during active treatment with VA before the patient discontinued treatment.

**Supplemental Table 2:** Summary of ADA levels for ADA-positive samples.

|  | Age (years) | Sex | IRR | Baseline ADA (U/mL) | Maximal ADA  on VA (U/mL) | Final ADA^b^ (U/mL) |
| --- | --- | --- | --- | --- | --- | --- |
| Pooled analysis group | 8 | F | Yes | <1.4 | 1012 | 1012 |
|  | 15 | M | Yes | <1.4 | 440 | 43.0 |
|  | 35 | F |  | <1.4 | 2.3 | <1.4 |
|  | 22^a^ | F |  | 1.4 | not evaluated | <1.4 |
|  | 15 | F |  | 2.1 | 2.0 | <1.4 |
|  | 12 | M |  | 3.1 | 3.6 | 2.7 |
|  | 9 | M |  | 1.8 | 2.3 | <1.4 |
|  | 12^a^ | M |  | <1.4 | not evaluated | <1.4 |
|  | 7 | M |  | 2.9 | 4.9 | <1.4 |
|  | 7^a^ | M |  | <1.4 | not evaluated | 24.0 |

ADA, antidrug antibody; F, female; IRR, infusion-related reaction; M, male VA, velmanase alfa.

^a^Three patients had only a single assessment of ADA, taken at the comprehensive evaluation visit in rhLAMAN-10 study (“final ADA”), as these patients were allocated in placebo arm in rhLAMAN-05 study and were then treated in the compassionate-use program.

^b^Final ADA is defined as last available ADA assessment. For patients with only one assessment of ADA taken at the comprehensive evaluation visit, this single assessment is categorized as “final ADA.”

**Supplemental Table 3:** ADA-positive patients by category.

|  | Total ADAs  (placebo or  VA-treated) | ADAs present  at baseline | Persistent treatment-emergent ADAs | Placebo-emergent  ADAs | ADAs with IRR |
| --- | --- | --- | --- | --- | --- |
| Pooled analysis group  N = 33  n (%) | 10 (30.3)^a,b,c,d^ | 5 (15.2) | 4 (12.1)^a,c,d^ | 1 (3.0)^b^ | 2 (6.1)^a^ |

ADA, antidrug antibody; IRR, infusion-related reaction; VA, velmanase alfa.

ADA-positive patients are those with values ≥ 1.4 U/mL in the rhLAMAN-10 study integrated analysis.

ADA-evaluable patients are patients with both baseline and ≥ 1 ADA test results on-treatment with VA or placebo.

**^a^**One patient was included in both the rhLAMAN-02 and the rhLAMAN-05 studies and, thus, was only counted once.

**^b^**One patient had ADA measurements of ≥ 1.4 U/mL during placebo treatment but not at baseline or during treatment with VA.

**^c^**One patient had only a single assessment of ADA, taken at the Comprehensive Evaluation Visit in the rhLAMAN-10 study (“final ADA”); this subject received placebo in the rhLAMAN-05 study.

**Supplemental Figure 1:** Serum oligosaccharides and serum IgG sensitivity analysis by ADA status.


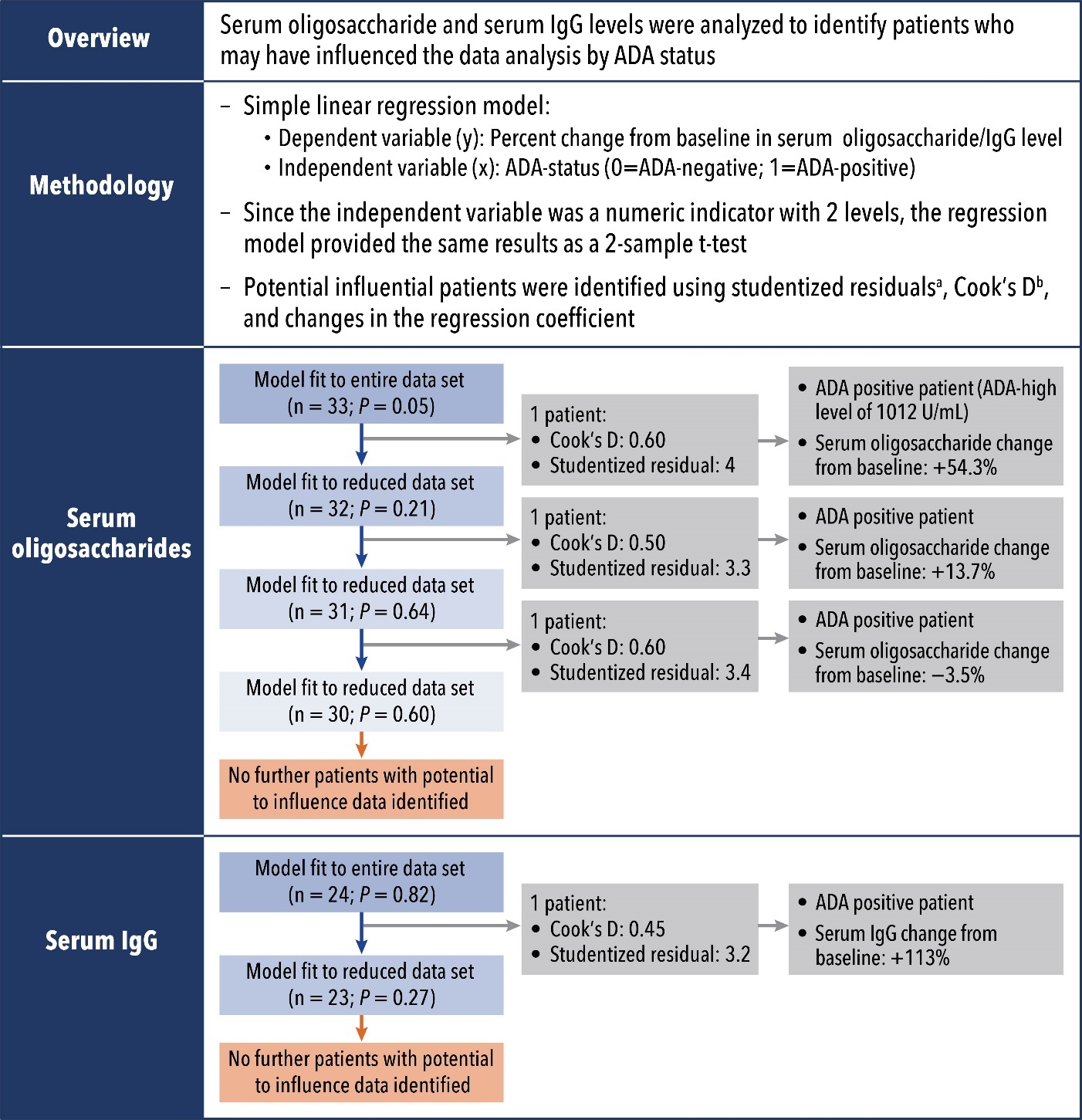


ADA, antidrug antibody; IgG, immunoglobulin G.

^a^Studentized residual is a standardized measure of the distance between the observation and the fitted regression line.

^b^Cook’s D is a combined measure of how the dependent and independent variables shape the regression. Value of 0.2 is the threshold for a “large” Cook’s D.
